# *PTCH1* Gene Mutations in Keratocystic Odontogenic Tumors: A Study of 43 Chinese Patients and a Systematic Review

**DOI:** 10.1371/journal.pone.0077305

**Published:** 2013-10-21

**Authors:** Yan-Yan Guo, Jian-Yun Zhang, Xue-Fen Li, Hai-Yan Luo, Feng Chen, Tie-Jun Li

**Affiliations:** 1 Department of Oral Pathology, Peking University School and Hospital of Stomatology, Beijing, China; 2 Central Laboratory, Peking University School and Hospital of Stomatology, Beijing, China; Indiana University School of Medicine, United States of America

## Abstract

**Background:**

The keratocystic odontogenic tumor (KCOT) is a locally aggressive cystic jaw lesion that occurs sporadically or in association with nevoid basal cell carcinoma syndrome (NBCCS). *PTCH1*, the gene responsible for NBCCS, may play an important role in sporadic KCOTs. In this study, we analyzed and compared the distribution pattern of *PTCH1* mutations in patients with sporadic and NBCCS-associated KCOTs.

**Methods:**

We detected *PTCH1* mutations in 14 patients with NBCCS-associated KCOTs and 29 patients with sporadic KCOTs by direct sequencing. In addition, five electronic databases were searched for studies detecting *PTCH1* mutations in individuals with NBCCS-associated or sporadic KCOTs, published between January 1996 and June 2013 in English language.

**Results:**

We identified 15 mutations in 11 cases with NBCCS-associated KCOTs and 19 mutations in 13 cases with sporadic KCOTs. In addition, a total of 204 *PTCH1* mutations (187 mutations from 210 cases with NBCCS-associated and 17 mutations from 57 cases with sporadic KCOTs) were compiled from 78 published papers.

**Conclusions:**

Our study indicates that mutations in transmembrane 2 (TM2) are closely related to the development of sporadic KCOTs. Moreover, for the early diagnosis of NBCCS, a genetic analysis of the *PTCH1* gene should be included in the new diagnostic criteria.

## Introduction

Originally described as cysts [1], odontogenic keratocysts (OKCs) were recategorized as neoplastic lesions under the name “karatocystic odontogenic tumors” (KCOTs) in the 2005 edition of the World Health Organization Classification of Head and Neck Tumors [2]. KCOTs are locally aggressive jaw cystic lesions that have putative growth potential and a propensity for recurrence [3], [4]. They may occur in isolation or in association with nevoid basal cell carcinoma syndrome (NBCCS, also known as Gorlin syndrome; OMIM No. 109400). NBCCS is a rare autosomal dominant disorder with an estimated prevalence of 1/55600 [5] to 1/256000 [6]. Affected patients present with a spectrum of developmental abnormalities, including palmar or plantar pits, calcification of the falx cerebri, bifid ribs, and an increased susceptibility to different neoplasms, such as multiple basal cell carcinomas (BCCs), KCOTs, medulloblastoma, and ovarian fibroma [7]–[9].

The human homolog of the *Drosophila* segment polarity gene *PTCH1* (OMIM No. 601309) has been identified as the gene responsible for NBCCS [10]–[12]. *PTCH1* has been mapped to 9q22.3-31 and consists of 23 coding exons spanning approximately 74 kb and encoding a 1447-amino-acid transmembrane (TM) glycoprotein. The Patched protein is involved in the Hedgehog (Hh) signaling pathway, a key regulator of body patterning and organ development during embryogenesis [13]. Patched and Smoothened (Smo) are thought to act as subunits of a putative Hh receptor complex: Smo functions as the transducing subunit, and its activity is blocked by a direct interaction with Patched, the ligand-binding subunit [14], [15]. Misregulation of the signaling caused by inactivation of *PTCH1* is involved in the development of NBCCS and some related sporadic tumors, supporting the hypothesis that *PTCH1* functions as a tumor suppressor gene [10]. Several studies have demonstrated the presence of *PTCH1* mutations in patients with NBCCS [12] as well as in some sporadic tumors, including BCCs [16], medulloblastoma [17], and KCOTs [18].

The Patched protein has several important functional domains. Two large extracellular loops (ECLs) are required for the binding of N-Shh to the Patched protein, and glycosylation sites in the two large ECLs are required for N-Shh binding [19]. Martin et al. (2001) and Strutt et al. (2001) suggested that the sterol-sensing domain (SSD) of Patched is required to negatively regulate Smoothened (Smo) activity, indicating a role of the SSD in mediating the vesicular trafficking of *PTCH* to regulate Smo activity [20], [21]. Additionally, its large intracellular loop (ICL) participates in a G_2_/M checkpoint by regulating the localization of M-phase promoting factor [22]. The C-terminal region of Patched has been shown to induce apoptotic cell death during spinal cord development [23].

The distribution pattern of *PTCH1* mutations has been analyzed in patients with NBCCS as well as in several tumors including BCCs, BCCs with xeroderma pigmentosum syndrome (XP-BCCs), and sporadic medulloblastomas [24]. They found that *PTCH1* mutations were identified throughout nearly the entire sequence, with a high frequency of mutations clustered in the two large extracellular loops and the large intracellular loop [24]. NBCCS cases and each class of tumor analyzed revealed a different distribution of mutations in the various Patched protein domains [24]. *PTCH1* gene harbors mutational hot spot regions, including a slippage-sensitive sequence in the N-terminus [24]. However, few reports have described the distribution pattern in KCOTs, especially sporadic KCOTs. Thus, the aim of the present study was to investigate *PTCH1* gene mutations in 29 sporadic and 14 NBCCS-associated KCOTs. Additionally, to systematically analyze and compare the distribution pattern of *PTCH1* mutations in NBCCS-associated and sporadic KCOTs, we conducted a systematic review of published studies evaluating *PTCH1* mutations in patients with KCOTs.

## Materials and Methods

### Subjects and Samples

KCOT samples from 43 unrelated Chinese patients (14 patients with NBCCS-related and 29 with sporadic KCOTs) were obtained from Peking University, School and Hospital of Stomatology (Beijing, China). Fresh KCOT tissue specimens and corresponding peripheral blood samples were collected and immediately stored at −80°C for subsequent polymerase chain reaction (PCR) and sequencing analysis. Peripheral blood control samples were obtained from 100 normal volunteers from the Blood Transfusion Center at Peking University First Hospital. The protocol for this study was approved by the Ethics Committee of Peking University's Health Science Center. Written informed consent was obtained from all study participants.

### DNA Extraction and PCR

Genomic DNA was extracted from the frozen tissue specimens and peripheral blood samples was extracted with a QIAamp DNA Mini Kit (Qiagen, Hilden, Germany), and 22 coding exons (exons 2–23) and the exon-intron boundaries of *PTCH1* were amplified by PCR with specific primers. Two primer sets were used separately to amplify exons 14 and 23 because of the relatively large size of the exons. PCR was performed in a final reaction volume of 50 µL, containing approximately 100 ng of template DNA, 200 µM dNTPs, 10 µM each primer and 1.25 U of Ex Taq DNA polymerase (Takara, Kyoto, Japan) in PCR buffer. Amplification was performed in a thermal cycler (Eppendorf, Hamburg, Germany) using the following cycling parameters: initial incubation at 94C for 5 min; 35 cycles of denaturation at 94°C for 30 s, annealing at 56–65°C for 30 s, and elongation at 72°C for 30 s; and final elongation at 72°C for 7 min.

### Mutation Analysis by Direct and Clonal Sequencing

The amplified products were sequenced directly with the primers used for the original PCR. Insertion or deletion mutations were confirmed by cloning purified PCR products into the plasmid vector pCR2.1 (Invitrogen, Carlsbad, CA, USA). After transforming competent *Escherichia coli strain* TOP10 with the recombinant vectors, white colonies were selected and grown overnight in 3 mL of LB medium with ampicillin. Sequencing was performed on an ABI PRISM 3100 Genetic Analyzer (Applied Biosystems, Foster City, CA, USA) with M13 universal primers. Identified mutations were confirmed by reverse sequencing and at least two additional independent PCR products.

### Restriction Enzyme Analysis

To define unreported *PTCH1* mutations (missense mutations, in particular) as novel mutations rather than rare polymorphisms, we tested 100 unrelated control DNAs by restriction enzyme analysis. Digestion of the PCR products with *Msp*I, *Ban*II or *BaeG*I (New England Biolabs, Beverly, MA, USA) was performed as recommended by the supplier. The digested DNA was electrophoresed on 2.5% agarose gels and stained with nucleic acid stain.

### Literature Review

We performed a systematic search from January 1996 to June 2013 using five electronic databases (PubMed, ScienceDirect, OviSP, Web of Science, and the Human Gene Mutation Database). Additional studies were identified by searching the reference lists of the included publications. The keywords used were “*PTCH1*” (or its synonyms) and “NBCCS or KCOT” (or their synonyms). This review was conducted and reported according to the PRISMA (Preferred Reporting Items for Systematic Reviews and Meta-Analyses) Statement issued in 2009 (Checklist S1) [25].

**Figure 1:**
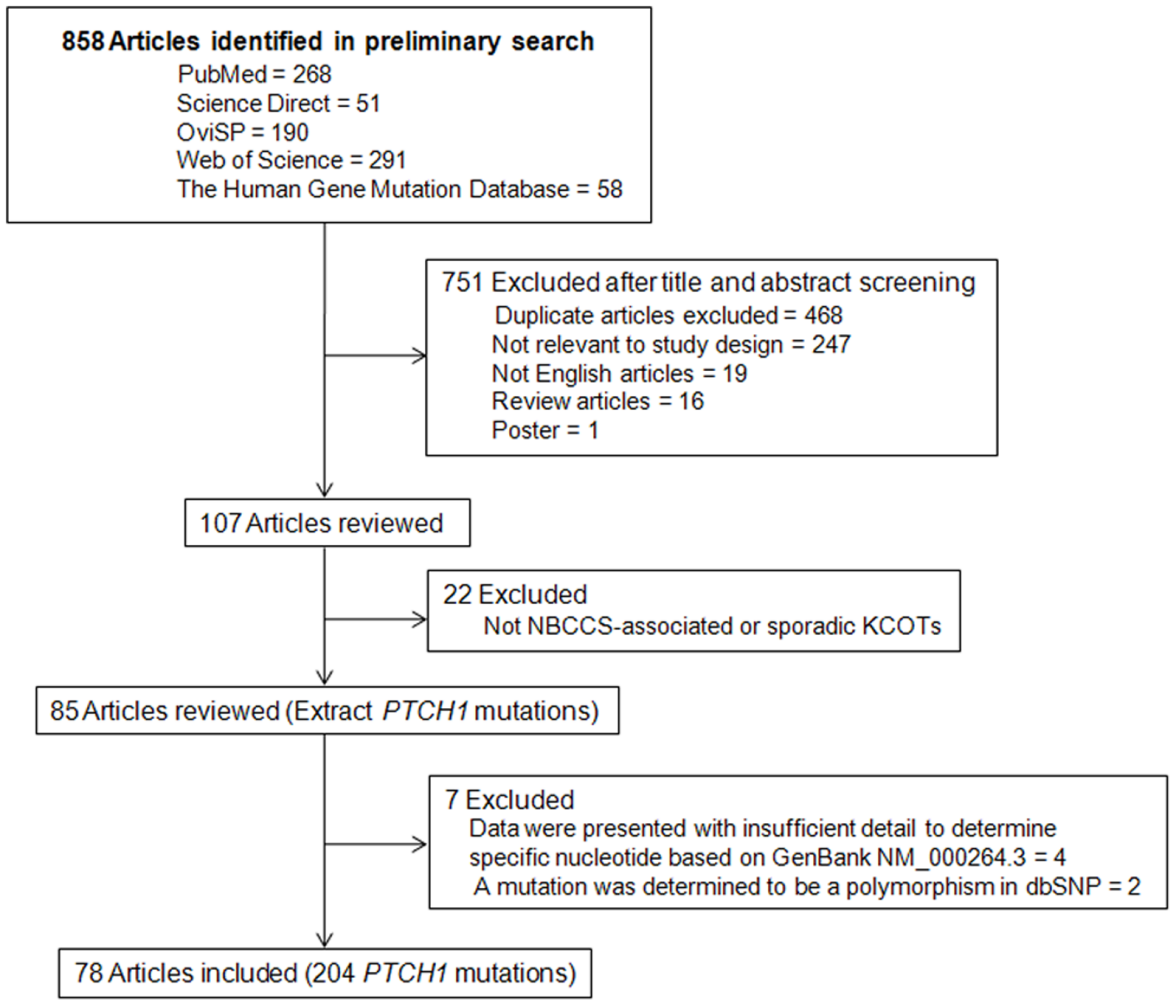
Flow chat of the selection process for the extensive review.

Studies were included if they concerned *PTCH1* gene mutations in patients with sporadic or NBCCS-associated KCOTs. A list of exclusionary indications is shown in Fig. 1. Only one entry was made when the same mutation in a single patient was reported in different papers (for example: c.2186A>T was first reported by Ponti et al. [26] and subsequently by Pastorino et al. [27]). Separate entries were made for each patient as recurrent mutations when the same mutation was reported in apparently unrelated patients (for example: c.403C>T identified in different patients [18], [28]). Nucleotide and protein names were given for each mutation according to current nomenclature guidelines (http://www.hgvs.org/mutnomen/). Nucleotide numbering was based on GenBank entry NM_000264.3, where the A of the ATG initiation codon represents nucleotide +1. The amino acid numbering was based on GenBank entry NP_000255.2.

### Statistical Analysis

Data were analyzed using *SPSS* software (ver. 13.0), and significant differences between categorical groups were determined using the chi-squared test or Fisher's exact test, while numerical groups were assessed using the independent *t*-test. *P* values<0.05 were considered to indicate statistical significance. All statistical tests were two-sided.

## Results

### Clinical Manifestations of Patients with NBCCS-associated KCOTs

**Figure 2:**
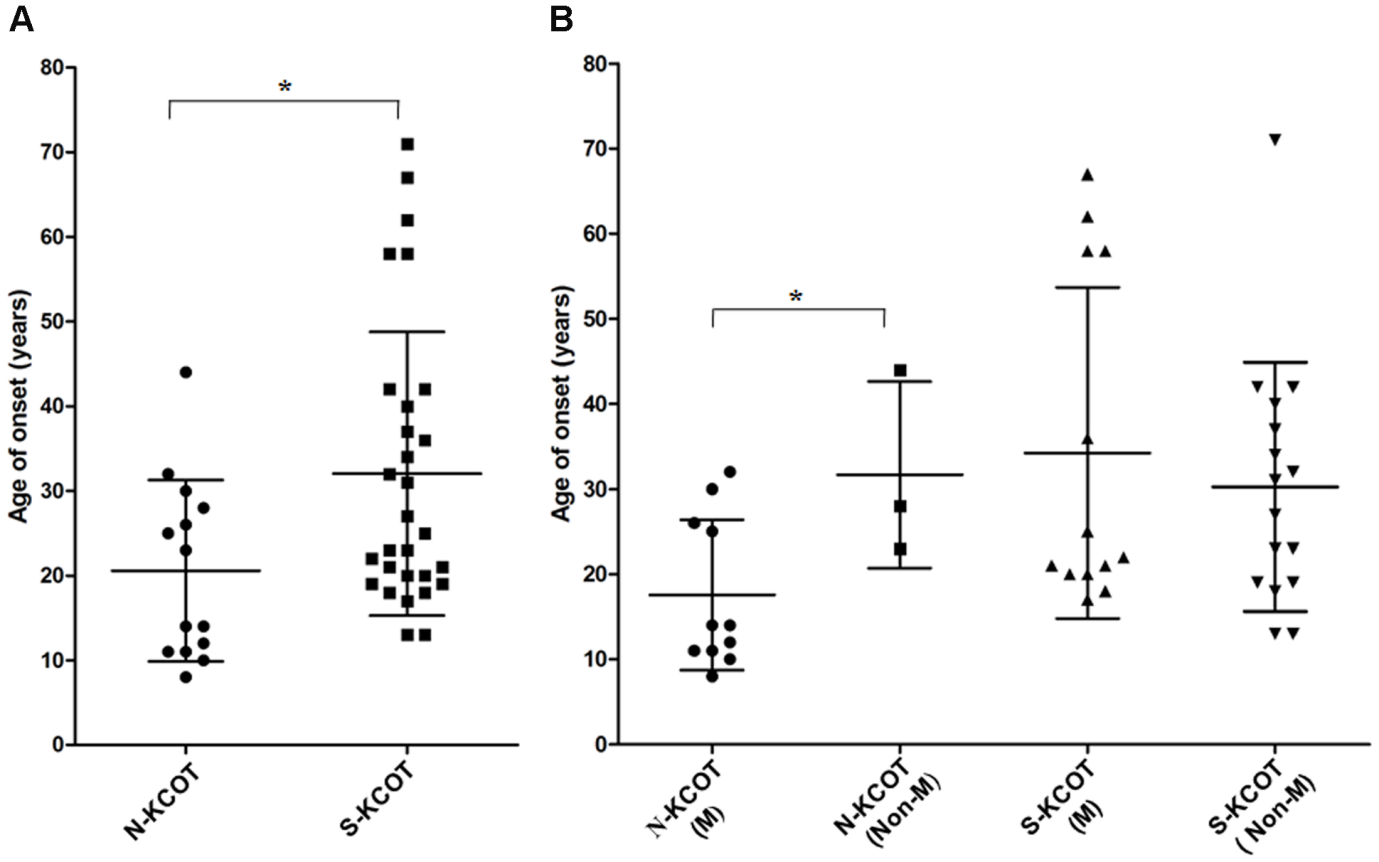
The onset age distribution of patients with NBCCS-associated and sporadic KCOTs. (A) The age distribution of 14 patients with syndromic KCOTs was significantly lower than in 29 patients with sporadic KCOTs (*P* = 0.025). (B) In syndromic KCOTs, the age distribution in the mutation group was significantly lower than in non-mutation group (*P* = 0.037), whereas in sporadic KCOTs, no significant difference was detected between the groups (*P* = 0.537). “N-KCOT” indicates “NBCCS-associated KCOT”; “S-KCOT” indicates “sporadic KCOT”; “M” indicates “Mutation group”; and “Non-M” indicates “Non-mutation group” (Error bars±SD, * *P*<0.05).

The male to female ratio among the 14 patients with NBCCS-associated KCOTs was 3.67∶1. To improve the comparability of age factors, we used age of onset (age at diagnosis minus duration) in the following statistical analysis. The individuals ranged in onset age from 8 to 44 years (mean 20.57±10.71; median 18.50 years; Fig. 2A).

The total number of lesions was 45, from 1 to 5, and the average number of lesions was 3.21 (Table 1). Of the lesions, 18/45 (40.0%) were located in the maxilla and 27/45 (60.0%) in the mandible. Among the 14 cases, only one patient had a single lesion of KCOT; however, the scope of the lesion in this patient was wide, approximately from the right mandibular angle to the left mandibular angle.

**Table 1:** Clinical manifestations and *PTCH1* mutations in 14 cases with NBCCS-associated KCOTs.

Patient	Sex/ Onset age	Number of Lesions		Nomenclature c.	Nomenclature p.	Mutation type	Exon/Intron no.	Characterization	Structure
		Maxilla	Mandible						
NB21^d^	M/11	2	3	c.233G>A	p.Trp78X	Missense mutations	Exon2	Germline	N-terminus
				c.3253dupA	p.Ile1085AsnfsX60	Small duplications	Exon19	Somatic	TM10
NB22	M/11	1	2	c.873C>G	p.Tyr291X	Nonsense mutations	Exon6	Germline	ECL1
				c.2479A>G^a^	p.Ser827Gly	Missense mutations	Exon15	Germline	ECL4
NB23	M/14	1	3	c.394+3_+20del18		Splice-site mutations	Intron2	Somatic	N-terminus
				c.1526G>A^b^	p.Gly509Asp	Missense mutations	Exon11	Germline	TM4
NB24^d^	M/14	1	2	c.3156_3163delins19	p.Ala1053LeufsX2	Indel mutations	Exon18	Somatic	ICL4
				c.2709_2713delTAAAC	p.Lys904AlafsX10	Small out-of-frame deletions	Exon17	Somatic	ECL4
NB25^d^	M/25	0	2	c.3180_3185del6	p.Ala1061_Leu1062del	Small in-frame deletions	Exon19	Germline	TM9
NB26	F/30	1	2	c.534_545del12	p.His178_Ala182delinsGln	Small in-frame deletions	Exon3	Germline	ECL1
NB27^d^	M/8	1	2	c.1342_1345delCTCA	p.Leu448CysfsX7	Small out-of-frame deletions	Exon9	Germline	TM2
NB28	M/12	2	2	c.584+1G>A		Splice-site mutations	Intron3	Germline	ECL1
NB29	M/32	1	1	c.1531G>C	p.Gly511Arg	Missense mutations	Exon11	Germline	TM4
NB30^d^	F/10	1	2	c.387G>A^c^	p.Trp129X	Nonsense mutations	Exon2	Germline	ECL1
NB31^d^	M/26	3	2	c.2464dupC	p.Leu822ProfsX7	Small duplications	Exon15	Germline	ECL4
NB32	M/28	0	1	Not identified					
NB33	M/23	1	2	Not identified					
NB34	F/44	3	1	Not identified					

a This mutation has been reported in a holoprosencephaly patient [40].

b This mutation has been reported in a NBCCS patient [41].

c This mutation has been reported in a NBCCS patient [42].

d Those patients suffered from cleft lip or plate.

Abbreviations: NB, NBCCS.

### Clinical Manifestations of Patients with Sporadic KCOTs

The male to female ratio among the 29 patients with sporadic KCOTs was 1.07∶1. The age range of the 29 affected persons was 13 to 71 years (mean 32.03±16.75; median 25.0 years; Fig. 2A). The mean age of patients with sporadic KCOTs was significantly higher than that of patients with systematic KCOTs (t = -2.333,*P* = 0.025). All the 29 cases had a single lesion (Table 2). Of 29 lesions, only 2 (6.90%) were located in the maxilla and 27 (93.10%) in the mandible.

**Table 2:** Clinical manifestations and *PTCH1* mutations in 29 cases with sporadic KCOTs.

Patient	Sex/ Onset age	Number of Lesions		Nomenclature c.	Nomenclature p.	Mutation type	Exon/Intron no.	Characterization	Structure
		Maxilla	Mandible						
KC43	F	0	1	c.1359_1362delCTGT	p.Cys454X	Small out-of-frame deletions	Exon10	Somatic	TM2
				c.1493_1494insTG	p.Thr499GlufsX44	Small in-frame insertions	Exon10	Somatic	ECL2
KC44	M	0	1	c.536T>C	p.Leu179Pro	Missense mutations	Exon3	Somatic	ECL1
				c.1164dupC	p.Glu389ArgfsX48	Small duplications	Exon8	Somatic	ECL1
KC45	M	0	1	c.2638_2668del31	p.Gly880ProfsX13	Small out-of-frame deletions	Exon16	Somatic	ECL4
				c.260_271delinsAA	p.Leu87X	Indel mutations	Exon2	Somatic	N-terminus
KC46	F	0	1	c.3346G>C	p.Val1116Leu	Missense mutations	Exon20	Somatic	ICL5
				c.1127_1143del17	p.Phe376CysfsX55	Small out-of-frame deletions	Exon8	Somatic	ECL1
KC47	M	0	1	c.202-3C>T		Splice-site mutations	Intron1	Somatic	N-terminus
				c.1362_1375del14	p.Cys454TrpfsX38	Small out-of-frame deletions	Exon10	Somatic	TM2
KC48	M	0	1	c.1327delG	p.Ala443ProfsX13	Small out-of-frame deletions	Exon9	Somatic	TM2
				c.1344_1347delCATG	p.Met449SerfsX6	Small out-of-frame deletions	Exon9	Somatic	TM2
KC49	M	1	0	c.407dupT	p.Ser137LysfsX3	Small duplications	Exon3	Somatic	ECL1
KC50	M	0	1	c.3081dupG	p.Leu1028AlafsX117	Small duplications	Exon18	Somatic	TM8
KC51	F	0	1	c.2430delA	p.Asp811ThrfsX19	Small out-of-frame deletions	Exon15	Somatic	ECL4
KC52	M	0	1	c.1063_1067+11del16		Splice-site mutations	Exon7/Intron7	Somatic	ECL1
KC53	F	0	1	c.2908G>T^a^	p.Glu970X	Nonsense mutations	Exon18	Somatic	ECL4
KC54	F	1	1	c.262_265delTTTA^b^	p.Phe88AsnfsX28	Small out-of-frame deletions	Exon2	Somatic	N-terminus
KC55	M	0	1	c.1394_1396delinsA	p.Ser465X	Indel mutations	Exon10	Somatic	ICL1
KC56	F	0	1	Not identified					
KC57	F	0	1	Not identified					
KC58	M	0	1	Not identified					
KC59	F	0	1	Not identified					
KC60	F	0	1	Not identified					
KC61	M	0	1	Not identified					
KC62	M	0	1	Not identified					
KC63	F	0	1	Not identified					
KC64	M	0	1	Not identified					
KC65	F	0	1	Not identified					
KC66	F	0	1	Not identified					
KC67	M	0	1	Not identified					
KC68	F	0	1	Not identified					
KC69	F	0	1	Not identified					
KC70	M	0	1	Not identified					
KC71	F	0	1	Not identified					

a This mutation has been reported in a NBCCS patient [34].

b This mutation has been reported in a NBCCS patient [43].

Abbreviations: KC, KCOT.

### *PTCH1* Mutations in Patients with NBCCS-associated and Sporadic KCOTs

We identified 11 germline and 4 somatic mutations in 11 of 14 (78.57%) patients with NBCCS-associated KCOTs (Table 1). Additionally, 12 mutations (80.0%) had not been previously reported in the literature and consisted of 7 truncation-causing, 3 non-truncation-causing, and 2 splice-site mutations. Of the 12 new mutations, 5 were distributed in the two large ECLs (ECL1 and ECL4), 4 in the TMs, 2 in the N-terminus, and 1 in the fourth ICL (ICL4). Furthermore, three cases (NB21, NB22, and NB23) were found to carry two concomitant mutations while NB24 was found to carry two somatic truncation-causing mutations (c.3156_3163delins19 and c.2709_2713delTAAAC), identified in different KCOTs. One mutation in patient NB21 was a germline truncation mutation in exon 2 (c.233G>A) and the other was a somatic truncation mutation in exon 19 (c.3253dupA), resulting in premature stops at codon 78 and 1144 respectively. Two germline mutations were identified in patient NB22, one was a truncation mutation in exon 6 (c.873C>G) and the other was a non-truncation mutation in exon 15 (c.2479A>G). Also, one mutation in patient NB23 was a germline non-truncation mutation in exon 11 (c.1526G>A) and the other was a somatic splice-site mutation in exon 19 (c.394+3_+20del18). Additionally, 6 of 14 cases (42.86%; NB21, NB24, NB25, NB27, NB30, and NB31) suffered from cleft lip or plate, an incidence significantly higher than that in previous studies [9], [29], [30]. All six cases were found to carry *PTCH1* mutations (Table 1). Patient NB25 carried a non-truncation mutation (c.3180_3185del6). One truncation mutation (c.1342_1345delCTCA) was identified in patient NB27. One truncation mutation (c.387G>A) was detected in patient NB30. Patient NB31 carried a truncation mutation (c.2464dupC) in the second large ECL (ECL2). In addition, A G>C substitution at nucleotide 1531 in exon 11, which resulted in a glycine to arginine substitution at codon 511, was detected in a 32-year-old male patient with NBCCS (NB29). This mutation was not detected in 100 control normal volunteers by direct sequencing.

The 14 affected patients could be divided into two groups: mutation and non-mutation. The mean age (17.55 years) of the mutation group was significantly younger than that (31.67 years) of the non-mutation group (Fig. 2B; t = −2.350, *P* = 0.037). All the patients under the age of 20 years carried mutations. Four patients over 20 years of age were identified to carry mutations whereas three patients over 20 years of age carried no mutations (Fig. 2B). Particularly, patient NB32, a 28-year-old patient with typical NBCCS phenotypes (such as multiple KCOTs and bilamellar calcification of the falx cerebri), was found to carry no *PTCH1* mutations and had only a single KCOT. Additionally, in the mutation group, 14 lesions were found in the maxilla while 23 in the mandible. In the non-mutation group, four lesions were found in the maxilla while five in the mandible (Table 1). No significant difference was detected between the two groups in terms of the lesion distribution (Pearson χ^2^ = 0.057,*P* = 0.811).

We identified 19 somatic *PTCH1* mutations in 13 of 29 (44.83%) patients with sporadic KCOTs (Table 2). No germline mutation was detected in any patient. Additionally, six cases (6/28, 21.43%) were found to carry two concomitant mutations. Of those mutations, 17 (89.47%) had not been previously reported, consisting of 13 truncation, 2 non-truncation, and 2 splice-site mutations. Of the 17 new mutations, seven were distributed in ECL1 and ECL4, five in TMs, two in N-terminus, one in the first intracellular loop (ICL1), one in ECL2 and one in ICL5. Additionally, novel missense mutations (c.536T>C and c.3346G>C) were respectively identified in patients KC44 and KC46. The two mutations introduced a novel restriction enzyme site for *Msp*I and *Ban*II, creating two fragments, thus confirming the presence of the mutation. To characterize the two novel missense mutations, we tested 100 control DNA samples from normal volunteers by restriction enzyme analysis. The abnormal restriction sites present in the PCR products from KC44 and KC46 were absent in the control DNAs. Thus, the two missense mutations were unlikely to be polymorphisms.

Similarly, the 28 affected cases could be further categorized into two groups: mutation and non-mutation. The mean age (34.23 years) of the mutation group was slightly higher than that (30.25 years) in non-mutation group (Fig. 2B), but the difference was not statistically significant (t = 0.630, *P* = 0.537). Moreover, only two cases (KC51 and KC54) occurred in the maxilla; both carried truncation mutation (c.2430delA and c.262_265delTTTA, respectively).

### Literature Search Results

Our search strategy retrieved 858 reports, 751 of which were excluded on the basis of the title and abstract. After carefully examining the full text of the remaining 107 articles, 78 articles (187 mutations from 210 cases with NBCCS-associated and 17 mutations from 57 cases with sporadic KCOTs) were included. Fig. 1 presents the flowchart of the search process. Tables S1-2 summarizes the key characteristics of each *PTCH1* mutation.

### Distribution Pattern of *PTCH1* Gene Mutations in Patients with Sporadic and NBCCS-associated KCOTs

Including the mutations found in our study, we compiled a list of 238 mutations (202 from patients with NBCCS-associated KCOTs and 36 from patients with sporadic KCOTs). Large deletions and duplications, although important in the pathogenesis of KCOTs, offered less detail in the distribution pattern of *PTCH1* mutations because those mutations encompassed more extensive structural changes. Thus, these 15 large deletions and duplications were excluded in the following statistical analysis.

**Figure 3:**
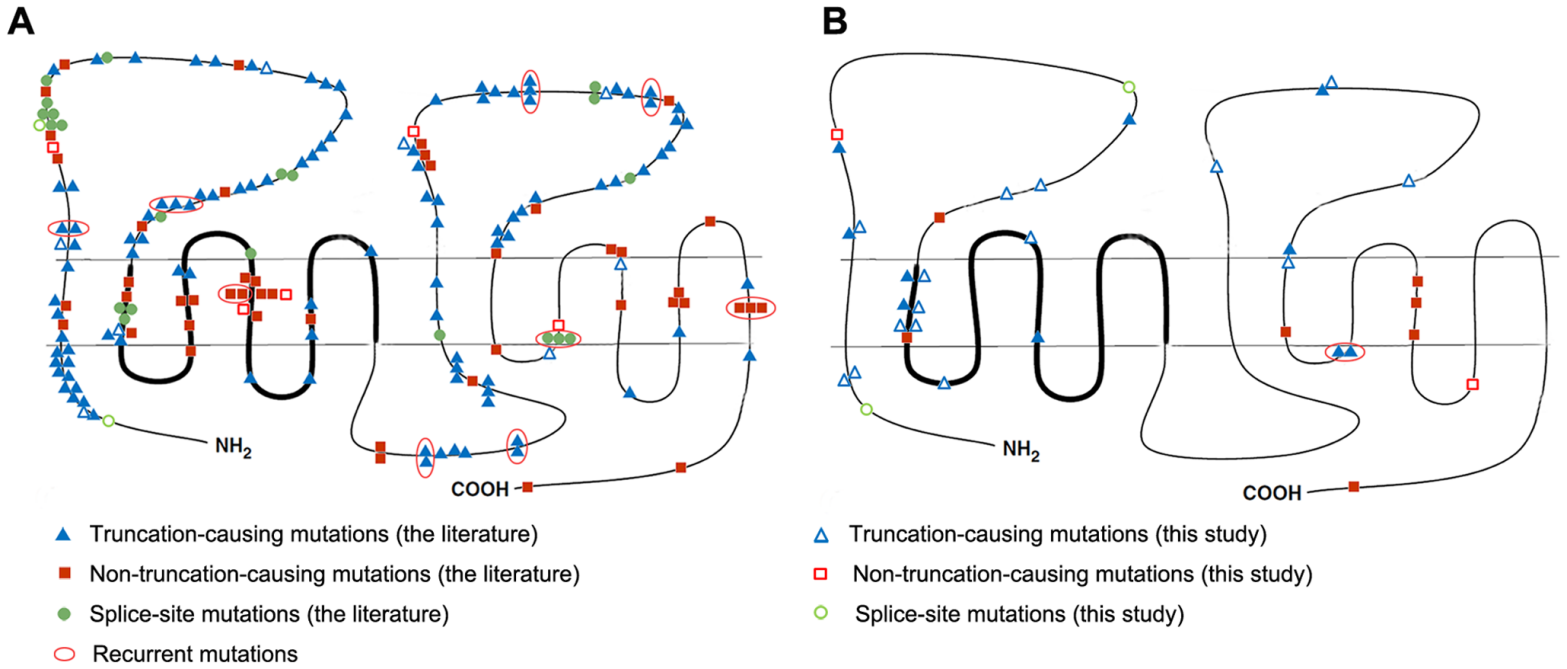
Distribution pattern of *PTCH1* mutations in relation to the different domains of the Patched protein. (A) NBCCS-associated KCOTs (n = 174). (B) Sporadic KCOTs (n = 36). The thick line indicates the SSD.

**Figure 4:**
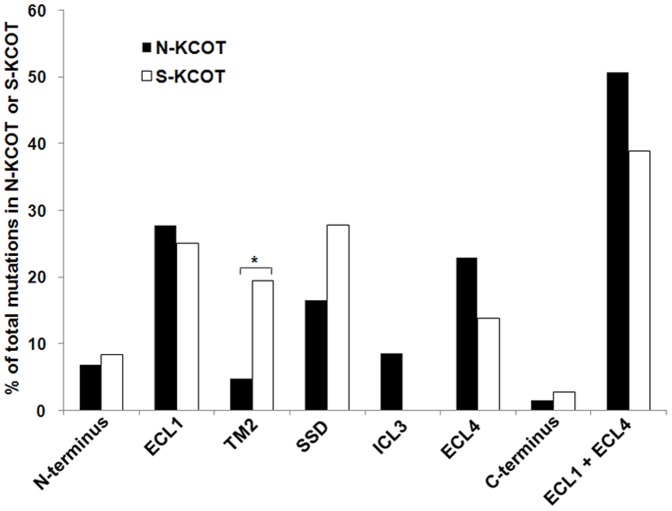
Proportion of *PTCH1* mutations in important domains of the Patched protein. ECL1 indicates the first large extracellular loop (amino acids 122–436); TM2 denotes the second transmembrane region (amino acids 437–457); SSD stands for the sterol-sensing domain (amino acids 438–598); ECL4 denotes the second large extracellular loop (amino acids 770–1027); * *P*<0.05.

Compared with the predicted Patched protein sequence, the 223 *PTCH1* mutations (187 from NBCCS-associated and 36 from sporadic KCOTs) were distributed over the entire sequence. The mutations (n = 187) from patients with NBCCS-associated KCOTs were mainly clustered in the two large extracellular loops (95/187, 50.80%; Fig. 3A and 4). Mutations were also found in the SSD (16.58%), the large intracellular loop (8.56%) and the N-terminal region (6.95%). Approximately half of the non-truncation-causing mutations (26/51, 50.98%) were located in the TM domains, especially TM4. Additionally, most splice-site mutations (11/23, 47.83%) were concentrated in the first large extracellular loops.

Mutations (n = 36) from patients with sporadic KCOTs were found predominantly in the two large extracellular loops (14/36, 38.89%) and TM2 (7/36, 19.44%; Fig. 3B and 4). Compared with the mutations in patients with NBCCS-associated KCOTs, the mutations from sporadic KCOTs were differently distributed, with a significantly higher frequency of mutations in TM2 (7/36 *vs*. 8/187; Pearson χ^2^ = 7.631,*P* = 0.006). Additionally, no mutation was found in the large intracellular loop (0/36), compared to those found in the NBCCS group (16/187; Pearson χ^2^ = 2.158, *P* = 0.142). Most non-truncation-causing mutations (5/9, 55.56%) were found in the TM domains, especially in the second half of the protein in patients with sporadic KCOTs.

### Different Mutation Types in Patients with Sporadic and NBCCS-associated KCOTs

The predominant mutation type seen in both the NBCCS-associated and sporadic KCOT groups was the predicted truncation-causing mutation in the Patched protein, with no statistically significant difference between the groups (113/187 *vs*. 25/36; Pearson χ^2^ = 1.040; *P* = 0.308; Table 3). Similarly, no significant difference in the distribution pattern of splice-site mutations or mutations that did not cause truncation of the Patched protein was observed between the groups.

**Table 3:** Different types of *PTCH1* mutations in patients with NBCCS-associated and sporadic KCOTs.

	Truncation		Non-truncation		Splice-site		Total
	No.	% of Total	No.	% of Total	No.	% of Total	
N-KCOT	113	61.49%	51	27.59%	23	10.92%	187
S-KCOT	25	69.44%	9	25.00%	2	5.56%	36
χ^2^ test	χ^2^ = 1.040		χ^2^ = 0.079		χ^2^ = 0.785		
	*P* = 0.308		*P* = 0.778		*P* = 0.375		

## Discussion

In the present study, 29 new *PTCH1* mutations were identified, including 20 truncation, 5 non-truncation, and 4 splice-site mutations. In general agreement with previous studies, most of the mutations identified were expected to lead to the synthesis of a truncated Patched protein. The *PTCH1* mutations were widely distributed along the entire sequence: 12 in the two large extracellular loops, 8 in the SSD, 4 in the N-terminus region, 3 in TM8-10, and 2 in ICL4 and ICL5. Those mutations enrich the spectrum of *PTCH1* mutations in KCOTs, thus providing further information about the pathogenesis of syndromic and sporadic KCOTs.

NBCCS is a hereditary condition caused by mutations in *PTCH1* gene and transmitted in an autosomal dominant manner with high penetrance and variable expressivity. In the present study, 10 of 14 (71.43%) individuals with syndromic KCOTs carried at least a germline *PTCH1* mutation. Because the initial tumor-causing mutation is inherited through the germline, it is already present in every cell of the body [31]. However, all 19 *PTCH1* mutations identified in sporadic KCOTs were somatic. Thus, it is not surprising that syndromic KCOTs presented at a younger age, and were multifocal with widespread sites of involvement located in the maxilla and mandible, whereas sporadic KCOTs presented at an older age, and were unifocal with local lesions mainly located in the mandible.

The mutation rate of *PTCH1* gene in NBCCS patients varied from 29.0 to 100% [32]–[34]. However, the mutation rate of *PTCH1* gene in sporadic KCOTs was approximately 28.6% [18]. Here, we identified 34 mutations in 11 of 14 (78.57%) syndromic and 13 of 29 (44.83%) sporadic KCOTs patients. Additionally, during the 14 cases with syndromic KCOTs, all patients under 20 years of age were detected to carry *PTCH1* mutations, whereas some patients over 20 years of age did not carry mutations. It indicated that *PTCH1* gene might play a more important role in the development of the younger age cases (≤20 years) with syndromic KCOTs, whereas the pathogenesis of the older age cases (>20 years) might be complicated and involved other as yet unknown genes. There were reports showing other genes, such as *PTCH2* [35] and *SUFU* [36], [37], might be involved in the pathogenesis of NBCCS. We suggested that KCOTs, as a complex disease, might be caused by multiple genes and environmental factors. To assess this, further study is needed to use other technologies, such as exome or whole-genome sequencing, to screen for additional genes.

Making a diagnosis of NBCCS in childhood patients could be challenging because several signs of the syndrome develop over time, including KCOTs, BCCs, calcification of the falx cerebri, and ovarian fibromas. Patients affected with NBCCS at birth might present only some congenital developmental deformities such as cleft lip or palate, and bifid, fused or accessory ribs. BCCs most often appear between puberty and 35 years of age; the mean age of onset is about 25 years of age [8]. Between 30% and 65% of patients with the syndrome have small asymmetric palmar or plantar pits by the age of 10 years; this percentage rises to 80% by the age of 15 years [7], [38]. The calcification of the falx cerebri can appear very early in life, and is often strikingly apparent from late childhood. Medulloblastoma characteristically presents during the first two years of life, while in the general population, occurrence peaks at 7 to 8 years of age. Ovarian fibromas generally develop in the teen years. In our study, two out of (14.3%) of patients develop a cystic lesion by the age of 10 years and 7 out of 14 (50%) by the age of 20 years. Diagnostic criteria were presently based on the most frequent and/or specific features of the syndrome [5], [9]. Thus, the diagnosis time of most NBCCS cases is usually postponed until the occurrence of symptoms. However, early diagnosis is often of great significance for the prevention and prognosis of systematic tumors. The high detection rate of *PTCH1* mutations among NBCCS patients, as demonstrated in the present study as well as other previous ones [34], [39], poses the possibility to include genetic analysis of *PTCH1* in the new diagnostic criteria for early diagnosis.

The distribution pattern of *PTCH1* mutations in NBCCS-associated and sporadic KCOTs had not been extensively analyzed previously. To our knowledge, this is the first to systematically analyze *PTCH1* mutation distribution pattern in systematic and sporadic KCOTs. Careful analysis of the two groups revealed several interesting findings. First of all, consistent with previous literature [24], *PTCH1* mutation hot regions in NBCCS-associated KCOTs involved the two large extracellular loops, sterol-sensing domain (SSD), large intracellular loop, and the N terminal region (close to TM1), while *PTCH1* mutation hot regions in sporadic KCOTs involved the two large extracellular loops and TM2. Additionally, compared to mutations in NBCCS-associated KCOTs, a significantly higher frequency of mutations in TM2 and no mutations in the large intracellular were found in sporadic KCOTs. Differences in the TM2 region were statistically significant, while the mutations in the large intracellular loop were not. Seven mutations in patients with sporadic KCOTs were distributed in the TM2 region, six of which resulted in premature truncation of Patched protein. Interestingly, a significantly lower frequency of mutations in TM2 was also noted in sporadic basal cell carcinomas and basal cell carcinomas with xeroderma pigmentosum syndrome [24]. Taken together, these results indicate that mutations in TM2 might be closely related to the development of sporadic KCOTs.

In conclusion, this is the first report to systematically analyze and compare the distribution patterns of *PTCH1* mutations in patients with NBCCS-associated and sporadic KCOTs, especially sporadic KCOTs. Our study indicates that mutations in TM2 are closely related to the development of sporadic KCOTs. Moreover, for the early diagnosis of NBCCS, genetic analysis of the *PTCH1* gene might be included in new diagnostic criteria. Our findings justify further investigation.

## Supporting Information

Table S1 Literature review: 187 *PTCH1* gene mutations in cases with NBCCS-associated KCOTs.
(DOCX)

Table S2 Literature review: 17 *PTCH1* gene mutations in cases with sporadic KCOTs.
(DOCX)

Checklist S1 **PRISMA Checklist for this systematic review.**
(DOC)
