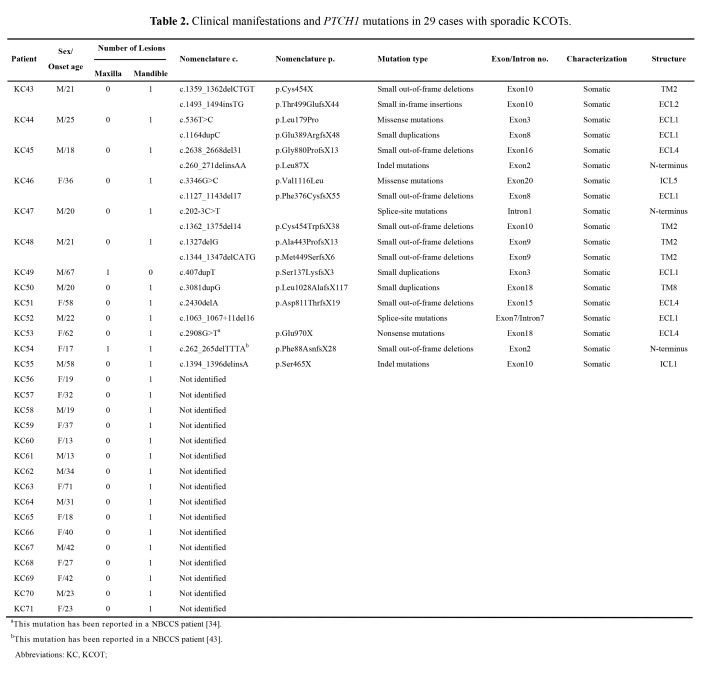# Correction: *PTCH1* Gene Mutations in Keratocystic Odontogenic Tumors: A Study of 43 Chinese Patients and a Systematic Review

**DOI:** 10.1371/annotation/6f3dfecd-6a37-44d3-9ee4-472d1eacc9a5

**Published:** 2014-01-02

**Authors:** Yan-Yan Guo, Jian-Yun Zhang, Xue-Fen Li, Hai-Yan Luo, Feng Chen, Tie-Jun Li

The onset age is missing from the second column in Table 2. Patient KC43 was also erroneously identified as female. Please see the corrected Table 2 here: 

**Figure:**