# Supplementary material for: PTCH1 Gene Mutations in Keratocystic Odontogenic Tumors: A Study of 43 Chinese Patients and a Systematic Review
Source: PLoS One. 2013 Oct 21;8(10):e77305. doi: 10.1371/journal.pone.0077305 (PMC3804548; doi:10.1371/journal.pone.0077305)
Supplement: Table S1 — Literature review: 187 PTCH1 gene mutations in cases with NBCCS-associated KCOTs. (DOCX) [file pone.0077305.s001.docx]

**Table S1.** Literature review: 187 *PTCH1* gene mutations in cases with NBCCS-associated KCOTs.

| **Mutation name** | **Patient** | **Nomenclature p.** | **Exon/intron  no.** | **Characterization** | **Structure** | **Reference no.** |
| --- | --- | --- | --- | --- | --- | --- |
| **Missense mutations** |  |  |  |  |  |  |
| c.317T>G | NB73 | p.Leu106Arg | Exon2 | Germline | TM1 | [1] |
| c.328G>T | NB98 | p.Gly110Trp | Exon2 | Germline | TM1 | [2] |
| c.688A>C | NB132 | p.Thr230Pro | Exon5 | Germline | ECL1 | [3] |
| c.709G>A | NB30 | p.Glu237Lys | Exon5 | Germline | ECL1 | [4] |
| c.863G>A | NB60 | p.Gly288Asp | Exon6 | Germline | ECL1 | [5] |
| c.1162A>T | NB161 | p.Asn388Tyr | Exon8 | Germline | ECL1 | [6] |
| c.1247C>G | NB61 | p.Thr416Ser | Exon9 | Somatic | ECL1 | [5] |
| c.1325T>A | NB53 | p.Val442Glu | Exon9 | Germline | TM2 | [7] |
| c.1346T>A | NB2 | p.Met449Lys | Exon9 | Germline | TM2 | [8] |
| c.1415C>A | NB88 | p.Ala472Asp | Exon10 | Germline | ICL1 | [9] |
| c.1436T>G | NB101 | p.Leu479Arg | Exon10 | Germline | TM3 | [10] |
| c.1451G>T | NB88 | p.Gly484Val | Exon10 | Germline | TM3 | [9] |
| c.1450G>A | NB121 | p.Gly484Arg | Exon10 | Germline | TM3 | [11] |
| c.1511C>T | NB122 | p.Pro504Leu | Exon11 | Germline | TM4 | [11] |
| c.1525G>C | NB110 | p.Gln509Arg | Exon11 | Germline | TM4 | [12] |
| c.1525G>C | NB203 | p.Gly509Arg | Exon11 | Germline | TM4 | [13] |
| c.1526G>T | NB102 | p.Gly509Val | Exon11 | Germline | TM4 | [10] |
| c.1526G>A | NB152 | p.Gly509Asp | Exon11 | Germline | TM4 | [14] |
| c.1660A>C | NB96 | p.Ser554Arg | Exon12 | Germline | TM5 | [2] |
| c.1939A>T | NB72 | p.Ser647Cys | Exon14 | Germline | ICL3 | [1] |
| c.1941C>A | NB123 | p.Ser647Arg | Exon14 | Germline | ICL3 | [11] |
| c.2186A>T | NB9 | p.Lys729Met | Exon14 | Germline | ICL3 | [15-17] |
| c.2465T>C | NB125 | p.Leu822Pro | Exon15 | Germline | ECL4 | [11] |
| c.2776T>C | NB128 | p.Trp926Arg | Exon17 | Germline | ECL4 | [11] |
| c.3062A>G | NB1 | p.Tyr1021Cys | Exon18 | Germline | ECL4 | [18] |
| c.3146A>T | NB40 | p.Asn1049Ile | Exon18 | Germline | ICL4 | [19] |
| c.3277G>C | NB8 | p.Glu1093Arg | Exon19 | Germline | TM10 | [16,20] |
| c.3394T>C | NB160 | p.Ser1132Pro | Exon20 | Germline | TM11 | [21] |
| c.3395C>A | NB206 | p.Ser1132Tyr | Exon20 | Germline | TM11 | [13] |
| c.3398C>T | NB67 | p.Thr1133Ile | Exon20 | Germline | TM11 | [22] |
| c.3440T>G | NB62 | p.Phe1147Cys | Exon20 | Germline | ECL6 | [5] |
| c.3499G>A | NB64 | p.Gly1167Arg | Exon21 | Germline | TM12 | [5] |
| c.3499G>A | NB107 | p.Gly1167Arg | Exon21 | Germline | TM12 | [10] |
| c.3499G>A | NB210 | p.Gly1167Arg | Exon21 | Germline | TM12 | [23] |
| c.3956G>A | NB88 | p.Arg1319His | Exon23 | Germline | C-terminus | [9] |
| c.4314G>T | NB186 | p.Glu1438Asp | Exon23 | Germline | C-terminus | [24] |
| **Silent mutations** |  |  |  |  |  |  |
| c.630C>T | NB4 | p.Ile1210Ile | Exon4 | Somatic | ECL1 | [25] |
| **Nonsense mutations** |  |  |  |  |  |  |
| c.250C>T | NB130 | p.Gln84X | Exon2 | Germline | N-terminus | [3] |
| c.279C>A | NB163 | p.Tyr93X | Exon2 | Germline | N-terminus | [26] |
| c.305T>A | NB131 | p.Leu102X | Exon2 | Germline | TM1 | [3] |
| c.387G>A | NB6 | p.Trp129X | Exon2 | Germline | ECL1 | [27] |
| c.403C>T | NB52 | p.Arg135X | Exon3 | Somatic | ECL1 | [28,29] |
| c.403C>T | NB176 | p.Arg135X | Exon3 | Germline | ECL1 | [30] |
| c.478C>T | NB84 | p.Gln160X | Exon3 | Germline | ECL1 | [31] |
| c.833G>A | NB87 | p.Trp278X | Exon6 | Germline | ECL1 | [9] |
| c.1012C>T | NB50 | p.Gln338X | Exon7 | Somatic | ECL1 | [28,29] |
| c.1018G>T | NB31 | p.Glu340X | Exon7 | Germline | ECL1 | [32] |
| c.1093C>T | NB202 | p.Gln365X | Exon8 | Germline | ECL1 | [33] |
| c.1138G>T | NB100 | p.Glu380X | Exon8 | Germline | ECL1 | [10] |
| c.1148C>G | NB77 | p.Ser383X | Exon8 | Germline | ECL1 | [34] |
| c.1196G>A | NB45 | p.Trp399X | Exon8 | Germline | ECL1 | [35] |
| c.1237C>T | NB21 | p.Gln413X | Exon9 | Germline | ECL1 | [16] |
| c.1585A>T | NB168 | p.Lys529X | Exon11 | Germline | ICL2 | [36] |
| c.1987C>T | NB169 | p.Gln663X | Exon14 | Germline | ICL3 | [36] |
| c.1987C>T | NB10 | p.Gln663X | Exon14 | Germline | ICL3 | [16] |
| c.2062C>T | NB175 | p.Gln688X | Exon14 | Germline | ICL3 | [30] |
| c.2062C>T | NB12 | p.Gln688X | Exon14 | Germline | ICL3 | [16] |
| c.2391C>A | NB79 | p.Tyr787X | Exon15 | Germline | ECL4 | [37] |
| c.2619C>A | NB58 | p.Tyr873X | Exon16 | Germline | ECL4 | [5] |
| c.2619C>A | NB70 | p.Tyr873X | Exon16 | Germline | ECL4 | [1,38] |
| c.2619C>A | NB126 | p.Tyr873X | Exon16 | Germline | ECL4 | [11] |
| c.2760C>A | NB34 | p.Tyr920X | Exon17 | Germline | ECL4 | [39] |
| c.2760C>A | NB210 | p.Tyr920X | Exon17 | Germline | ECL4 | [23] |
| c.2833C>T | NB170 | p.Arg945X | Exon17 | Germline | ECL4 | [40] |
| c.2908G>T | NB93 | p.Glu970X | Exon18 | Germline | ECL4 | [2] |
| c.3058C>T | NB34 | p.Gln1020X | Exon18 | Germline | ECL4 | [39] |
| **Splice-site mutations** |  |  |  |  |  |  |
| c.584+2T>G | NB33 |  | Intron3 | Germline | ECL1 | [39] |
| c.585-1G>A | NB18 |  | Intron3 | Germline | ECL1 | [16] |
| c.654+2T>A | NB11 |  | Intron4 | Germline | ECL1 | [16] |
| c.655-2A>T | NB108 |  | Intron4 | Germline | ECL1 | [10] |
| c.746+3_+4delAA | NB117 |  | Intron5 | Germline | ECL1 | [11] |
| c.747-1G>C | NB3 |  | Intron5 | Germline | ECL1 | [25] |
| c.945+1G>C | NB133 |  | Intron6 | Germline | ECL1 | [3] |
| c.1067+1G>C | NB49 |  | Intron7 | Germline | ECL1 | [41] |
| c.1068-2A>C | NB164 |  | Intron7 | Germline | ECL1 | [40] |
| c.1347+6G>A | NB57 | p.Val406_Gln501del | Intron9 | Germline | ECL1 | [5] |
| c.1348-2A>G | NB7 |  | Intron9 | Germline | TM2 | [16,42] |
| c.1504-2_1504-13del12 | NB135 |  | Intron10 | Germline | ECL2 | [3] |
| c.1504-6_-7ins21 | NB165 |  | Intron10 | Germline | TM2 | [40] |
| c.1504-1G>A | NB58 | p.Leu450_Glu534del | Intron10 | Somatic | TM2 | [5] |
| c.2560+1G>T | NB59 | p.Val751AspfsX49 | Intron15 | Germline | TM7 | [5] |
| c.2704-3T>C | NB76 |  | Intron16 | Germline | ECL4 | [43] |
| c.2887+1G>C | NB205 |  | Intron17 | Germline | ECL4 | [13] |
| c.3168+5G>A | NB140 |  | Intron18 | Germline | ECL4 | [3] |
| c.3169-2A>G | NB166 |  | Intron18 | Germline | TM9 | [40] |
| c.3169-2A>G | NB174 |  | Intron18 | Germline | TM9 | [30] |
| c.3169-2A>G | NB156 |  | Intron18 | Germline | TM9 | [44] |
| **Indel mutations** |  |  |  |  |  |  |
| c.260_264delinsAA | NB114 | p.Leu87X | Exon2 | Germline | N-terminus | [11] |
| c.742_743delinsGGAG | NB116 | p.Leu248GlyfsX2 | Exon5 | Germline | ECL1 | [11] |
| c.1515_1518delinsGAGA | NB136 | p.Phe505_Leu506delinsLeuArg | Exon11 | Germline | TM4 | [3] |
| c.2785_2806delins9 | NB94 | p.Asn929CysfsX29 | Exon17 | Germline | ECL4 | [2] |
| **Small out-of-frame deletions** |  |  |  |  |  |  |
| c.220delA | NB129 | p.Ala75ArgfsX5 | Exon2 | Germline | N-terminus | [3] |
| c.239_240delGA | NB43 | p.Arg80SerfsX9 | Exon2 | Germline | N-terminus | [35] |
| c.256_257delCT | NB177 | p.Leu87IlefsX2 | Exon2 | Germline | N-terminus | [30] |
| c.265_287del23 | NB198 | p.Gly91ArgfsX41 | Exon2 | Germline | N-terminus | [13] |
| c.278delA | NB82 | p.Tyr93SerfsX24 | Exon2 | Germline | N-terminus | [45] |
| c.331delG | NB71 | p.Ala111ProfsX6 | Exon2 | Germline | TM1 | [1] |
| c.479_482delAGAC | NB76 | p.Gln160ProfsX10 | Exon3 | Germline | ECL1 | [43] |
| c.768_777del10 | NB85 | p.Asn258LeufsX8 | Exon6 | Germline | ECL1 | [31] |
| c.808delA | NB167 | p.Ile271X | Exon6 | Germline | ECL1 | [46] |
| c.865delC | NB97 | p.His289MetfsX35 | Exon6 | Germline | ECL1 | [2] |
| c.912delC | NB153 | p.Ala306ProfsX18 | Exon6 | Germline | ECL1 | [14] |
| c.941delC | NB179 | p.Lys315AsnfsX9 | Exon6 | Germline | ECL1 | [30] |
| c.980delG | NB37 | p.Cys327PhefsX5 | Exon7 | Germline | ECL1 | [39] |
| c.1208_1209delAT | NB118 | p.Tyr403CysfsX33 | Exon8 | Germline | ECL1 | [11] |
| c.1208_1209delAT | NB119 | p.Tyr403CysfsX33 | Exon8 | Germline | ECL1 | [11] |
| c.1208_1209delAT | NB120 | p.Tyr403CysfsX33 | Exon8 | Germline | ECL1 | [11] |
| c.1291delC | NB55 | p.Leu431X | Exon9 | Germline | ECL1 | [11] |
| c.1472delT | NB39 | p.Ile491ThrfsX51 | Exon10 | Germline | TM3 | [47] |
| c.1615delG | NB137 | p.Glu539SerfsX3 | Exon12 | Germline | ICL2 | [3] |
| c.1670_1671delCA | NB46 | p.Thr557SerfsX69 | Exon12 | Germline | TM5 | [35] |
| c.2029delA | NB47 | p.Thr667ProfsX16 | Exon14 | Germline | ICL3 | [35] |
| c.2011delC | NB155 | p.His671ThrfsX22 | Exon14 | Germline | ICL3 | [14] |
| c.2178delC | NB4 | p.Cys727ValfsX19 | Exon14 | Germline | ICL3 | [25] |
| c.2179delT | NB50 | p.Cys727ValfsX19 | Exon14 | Germline | ICL3 | [28,29] |
| c.2195_2196delTC | NB180 | p.Ser733IlefsX4 | Exon14 | Germline | ICL3 | [30] |
| c.2196_2197delCT | NB60 | p.Ser733IlefsX4 | Exon14 | Germline | ICL3 | [5] |
| c.2197_2198delTC | NB3 | p.Ser733IlefsX4 | Exon14 | Germline | ICL3 | [25] |
| c.2287delG | NB138 | p.Val763SerfsX9 | Exon15 | Germline | TM7 | [3] |
| c.2395delT | NB162 | p.Ser799LeufsX7 | Exon15 | Germline | ECL4 | [26] |
| c.2442_2452del11 | NB201 | p.Ile815ThrfsX10 | Exon15 | Germline | ECL4 | [48] |
| c.2577_2583del7 | NB104 | p.Phe859LeufsX42 | Exon16 | Germline | ECL4 | [10] |
| c.2576_2582del7 | NB139 | p.Phe859LeufsX42 | Exon16 | Germline | ECL4 | [3] |
| c.2582delG | NB159 | p.Ser861MetfsX14 | Exon16 | Germline | ECL4 | [49] |
| c.2595delC | NB181 | p.Ile868SerfsX35 | Exon16 | Germline | ECL4 | [30] |
| c.2719delC | NB173 | p.Leu907TrpfsX17 | Exon17 | Germline | ECL4 | [30] |
| c.2798delC | NB89 | p.Ala933GlyfsX29 | Exon17 | Germline | ECL4 | [9] |
| c.2824delC | NB52 | p.Arg942GlyfsX20 | Exon17 | Germline | ECL4 | [28,29] |
| c.2869delC | NB56 | p.Pro957LeufsX5 | Exon17 | Germline | ECL4 | [50] |
| c.3000_3007del8 | NB183 | p.Asn1000LysfsX142 | Exon18 | Germline | ECL4 | [24] |
| c.3016delC | NB95 | p.Leu1006CysfsX43 | Exon18 | Germline | ECL4 | [2] |
| c.3042delC | NB127 | p.Phe1015SerfsX34 | Exon18 | Germline | ECL4 | [11] |
| c.3050_3051delTC | NB106 | p.F1017LfsX1143 | Exon18 | Germline | ECL4 | [2] |
| c.3375delC | NB141 | p.Val1126SerfsX13 | Exon20 | Germline | TM11 | [3] |
| c.3475delC | NB32 | p.Leu1159SerfsX32 | Exon21 | Germline | TM12 | [51] |
| c.3570delG | NB185 | p.Leu1190X | Exon22 | Germline | C-terminus | [24] |
| **Small in-frame deletions** |  |  |  |  |  |  |
| c.531_533delACA | NB115 | p.Gln177delinsHis | Exon3 | Germline | ECL1 | [11] |
| c.1537_1539delGAT  or c.1540_1542delGAT | NB68 | p.Asp513del  or p.Asp514del | Exon11 | Germline | TM4 | [52] |
| c.2444_2461del18 | NB74 | p.Ile815_Asp821delinsAsn | Exon15 | Germline | ECL4 | [53] |
| c.2446_2448delCAG | NB204 | p.Gln816del | Exon15 | Germline | ECL4 | [13] |
| c.3000_3005del6 | NB105 | p.Asn1000_Thr1002delinsLys | Exon18 | Germline | ECL4 | [10] |
| c.3244_3249del6 | NB199 | p.Pro1082_Val1083del | Exon19 | Germline | ECL5 | [13] |
| **Small out-of-frame insertions** |  |  |  |  |  |  |
| c.264_265insAATA | NB39 | p.Lys89AsnfsX52 | Exon2 | Somatic | N-terminus | [47] |
| c.282_283insA | NB178 | p.Gln95ThrfsX45 | Exon2 | Germline | N-terminus | [30] |
| c.323_324insGA | NB99 | p.Ile108MetfsX10 | Exon2 | Germline | TM1 | [10] |
| c.361_362insGAGC | NB69 | p.Leu121ArgfsX20 | Exon2 | Somatic | TM1 | [1] |
| **Small in-frame insertions** |  |  |  |  |  |  |
| c.1338_1339insGCG | NB71 | p.Tyr446_Leu447insAla | Exon9 | Germline | TM2 | [1,38] |
| **Small duplications** |  |  |  |  |  |  |
| c.290dupA | NB29 | p.Asn236LysfsX43 | Exon2 | Germline | ECL1 | [54] |
| c.705dupC | NB182 | p.Trp236LeufsX16 | Exon5 | Germline | ECL1 | [24] |
| c.931dupA | NB20 | p.Asn312LysfsX7 | Exon6 | Germline | ECL1 | [15,16] |
| c.1027_1028dupGT | NB134 | p.Gly344ThrfsX24 | Exon7 | Germline | ECL1 | [3] |
| c.1043_1046dupAGAA | NB86 | p.Asn349LysfsX88 | Exon7 | Germline | ECL1 | [55] |
| c.1138_1195dup58 | NB44 | p.Trp399X | Exon8 | Germline | ECL1 | [35] |
| c.1259dupT | NB154 | p.Ser421PhefsX16 | Exon9 | Germline | ECL1 | [56] |
| c.1261dupT | NB151 | p.Ser421PhefsX16 | Exon9 | Germline | ECL1 | [14] |
| c.1349dupT | NB112 | p.Ala451ArgfsX46 | Exon10 | Germline | TM2 | [57] |
| c.1370dupT | NB83 | p.Met457IlefsX40 | Exon10 | Germline | TM2 | [58] |
| c.1467dupA | NB158 | p.Leu490IlefsX7 | Exon10 | Germline | TM3 | [59] |
| c.1651dupA | NB172 | p.Thr551AsnfsX76 | Exon12 | Germline | TM5 | [30] |
| c.1723dupC | NB171 | p.Leu575ProfsX52 | Exon12 | Germline | ECL3 | [30] |
| c.2011_2012dupCA | NB103 | p.His673ArgfsX21 | Exon14 | Germline | ICL3 | [10] |
| c.2178dupC | NB124 | p.Cys727LeufsX17 | Exon14 | Germline | ICL3 | [11] |
| c.2256_2262dup7 | NB111 | p.Phe755GlyfsX37 | Exon15 | Germline | TM7 | [60] |
| c.2392dupC | NB157 | p.Phe798LeufsX31 | Exon15 | Germline | ECL4 | [59] |
| c.2454dupA | NB92 | p.Leu819ThrfsX10 | Exon15 | Germline | ECL4 | [2] |
| c.2535dupG | NB109 | p.His846ArgfsX15 | Exon15 | Germline | ECL4 | [61] |
| c.2712dupA | NB36 | p.Gln905ThrfsX11 | Exon17 | Germline | ECL4 | [39] |
| c.2795_2799dup5 | NB200 | p.Tyr934SerfsX30 | Exon17 | Germline | ECL4 | [13] |
| c.2918_2925dup8 | NB90 | p.Phe976SerfsX22 | Exon18 | Germline | ECL4 | [9] |
| c.3026dupA | NB184 | p.Tyr1009X | Exon18 | Germline | ECL4 | [24] |
| c.3244_3246dupCCC | NB63 | p.Pro1082dup | Exon19 | Germline | ECL5 | [5] |
| c.3325_3328dupGGCG | NB48 | p.Asp1110GlyfsX36 | Exon20 | Germline | ICL5 | [41] |
| **Large deletions** |  |  |  |  |  |  |
| del8.28Mb | NB25 |  | Exon1-23 | Germline |  | [16] |
| del8.07Mb | NB26 |  | Exon1-23 | Germline |  | [16] |
| del1.08Mb | NB27 |  | Exon1-23 | Germline |  | [16] |
| del15.33_16.04Mb | NB41 |  | Exon1-23 | Germline |  | [62] |
| del18.08_18.54Mb | NB42 |  | Exon1-23 | Germline |  | [62] |
| del1.2Mb | NB207 |  | Exon1-23 | Germline |  | [35] |
| del4.5Mb | NB54 |  | Exon1-23 | Germline |  | [63] |
| del7.7Mb | NB78 |  | Exon1-23 | Germline |  | [64] |
| del11.0Mb | NB80 |  | Exon1-23 | Germline |  | [65] |
| del165kb | NB81 |  | Exon1-23 | Germline |  | [65] |
| del | NB91 |  |  | Germline |  | [66] |
| del15.3_15.6Mb | NB113 |  | Exon1-23 | Germline |  | [67] |
| del | NB149 |  | Exon1-23 | Germline |  | [68] |
| del | NB150 |  |  | Germline |  | [69] |
| **Large duplications** |  |  |  |  |  |  |
| g.39362_58175dup18814^a^ | NB28 |  | Exon11-17 | Germline |  | [70] |

^a^Note that the mutation g.39362_58175dup18814 was given according to the genomic reference sequence (NG_007664.1).

**References**

1. Li TJ, Yuan JW, Gu XM, Sun LS, Zhao HS (2008) PTCH germline mutations in Chinese nevoid basal cell carcinoma syndrome patients. Oral Dis 14: 174-179.

2. Tanioka M, Takahashi K, Kawabata T, Kosugi S, Murakami K, et al. (2005) Germline mutations of the PTCH gene in Japanese patients with nevoid basal cell carcinoma syndrome. Arch Dermatol Res 296: 303-308.

3. Savino M, d'Apolito M, Formica V, Baorda F, Mari F, et al. (2004) Spectrum of PTCH mutations in Italian nevoid basal cell-carcinoma syndrome patients: identification of thirteen novel alleles. Hum Mutat 24: 441.

4. Zhang T, Chen M, Lu Y, Xing Q, Chen W (2011) A novel mutation of the PTCH1 gene activates the Shh/Gli signaling pathway in a Chinese family with nevoid basal cell carcinoma syndrome. Biochem Biophys Res Commun 409: 166-170.

5. Sun LS, Li XF, Li TJ (2008) PTCH1 and SMO gene alterations in keratocystic odontogenic tumors. J Dent Res 87: 575-579.

6. Ogata K, Ikeda M, Miyoshi K, Yamamoto Y, Yamamoto T, et al. (2001) Naevoid basal cell carcinoma syndrome with a palmar epidermoid cyst, milia and maxillary cysts. Br J Dermatol 145: 508-509.

7. Nakamura M, Tokura Y (2009) A novel missense mutation in the PTCH1 gene in a premature case of nevoid basal cell carcinoma syndrome. Eur J Dermatol 19: 262-263.

8. Kitano H, Koyama Y, Komiya M, Sato N, Nakayama T (2013) Basal cell nevus syndrome: New mutation of the patched homologue 1 gene. Journal of Oral and Maxillofacial Surgery, Medicine, and Pathology (in press).

9. Matsuzawa N, Nagao T, Shimozato K, Niikawa N, Yoshiura KI (2006) Patched homologue 1 mutations in four Japanese families with basal cell nevus syndrome. J Clin Pathol 59: 1084-1086.

10. Pastorino L, Cusano R, Nasti S, Faravelli F, Forzano F, et al. (2005) Molecular characterization of Italian nevoid basal cell carcinoma syndrome patients. Hum Mutat 25: 322-323.

11. Boutet N, Bignon YJ, Drouin-Garraud V, Sarda P, Longy M, et al. (2003) Spectrum of PTCH1 mutations in French patients with Gorlin syndrome. J Invest Dermatol 121: 478-481.

12. Ng D, Stavrou T, Liu L, Taylor MD, Gold B, et al. (2005) Retrospective family study of childhood medulloblastoma. Am J Med Genet A 134: 399-403.

13. Chidambaram A, Goldstein AM, Gailani MR, Gerrard B, Bale SJ, et al. (1996) Mutations in the human homologue of the Drosophila patched gene in Caucasian and African-American nevoid basal cell carcinoma syndrome patients. Cancer Res 56: 4599-4601.

14. Fujii K, Kohno Y, Sugita K, Nakamura M, Moroi Y, et al. (2003) Mutations in the human homologue of Drosophila patched in Japanese nevoid basal cell carcinoma syndrome patients. Hum Mutat 21: 451-452.

15. Ponti G, Pastorino L, Pollio A, Nasti S, Pellacani G, et al. (2012) Ameloblastoma: a neglected criterion for nevoid basal cell carcinoma (Gorlin) syndrome. Fam Cancer 11: 411-418.

16. Pastorino L, Pollio A, Pellacani G, Guarneri C, Ghiorzo P, et al. (2012) Novel PTCH1 mutations in patients with keratocystic odontogenic tumors screened for nevoid basal cell carcinoma (NBCC) syndrome. PLoS One 7: e43827.

17. Ponti G, Pollio A, Mignogna MD, Pellacani G, Pastorino L, et al. (2012) Unicystic ameloblastoma associated with the novel K729M PTCH1 mutation in a patient with nevoid basal cell carcinoma (Gorlin) syndrome. Cancer Genet 205: 177-181.

18. Torrelo A, Hernandez-Martin A, Bueno E, Colmenero I, Rivera I, et al. (2013) Molecular evidence of type 2 mosaicism in Gorlin syndrome. Br J Dermatol (in press).

19. Li J, Wang J, Liu Y, Wang W (2010) Analysis of mutation in exon 17 of PTCH in patients with nevoid basal cell carcinoma syndrome. Mol Biol Rep 37: 359-362.

20. Ponti G, Pollio A, Pastorino L, Pellacani G, Magnoni C, et al. (2012) Patched homolog 1 gene mutation (p.G1093R) induces nevoid basal cell carcinoma syndrome and non-syndromic keratocystic odontogenic tumors: A case report. Oncol Lett 4: 241-244.

21. Reifenberger J, Arnold N, Kiechle M, Reifenberger G, Hauschild A (2001) Coincident PTCH and BRCA1 germline mutations in a patient with nevoid basal cell carcinoma syndrome and familial breast cancer. J Invest Dermatol 116: 472-474.

22. Otsubo S, Honma M, Asano K, Takahashi H, Iizuka H (2008) A novel germ-line mutation of PTCH1 gene in a Japanese family of nevoid basal cell carcinoma syndrome: are the palmoplantar pits associated with true basal cell carcinoma? J Dermatol Sci 51: 144-146.

23. Barreto DC, Gomez RS, Bale AE, Boson WL, De Marco L (2000) PTCH gene mutations in odontogenic keratocysts. J Dent Res 79: 1418-1422.

24. Lench NJ, Telford EA, High AS, Markham AF, Wicking C, et al. (1997) Characterisation of human patched germ line mutations in naevoid basal cell carcinoma syndrome. Hum Genet 100: 497-502.

25. Kadlub N, Coudert A, Gatibelza ME, El Houmami N, Soufir N, et al. (2013) PTCH1 mutation and local aggressiveness of odontogenic keratocystic tumors in children: is there a relationship? Hum Pathol 44: 1071-1078.

26. Minami M, Urano Y, Ishigami T, Tsuda H, Kusaka J, et al. (2001) Germline mutations of the PTCH gene in Japanese patients with nevoid basal cell carcinoma syndrome. J Dermatol Sci 27: 21-26.

27. Suzuki M, Hatsuse H, Nagao K, Takayama Y, Kameyama K, et al. (2012) Selective haploinsufficiency of longer isoforms of PTCH1 protein can cause nevoid basal cell carcinoma syndrome. J Hum Genet 57: 422-426.

28. Pan S, Li TJ (2009) PTCH1 mutations in odontogenic keratocysts: are they related to epithelial cell proliferation? Oral Oncol 45: 861-865.

29. Pan S, Xu LL, Sun LS, Li TJ (2009) Identification of known and novel PTCH mutations in both syndromic and non-syndromic keratocystic odontogenic tumors. Int J Oral Sci 1: 34-38.

30. Wicking C, Gillies S, Smyth I, Shanley S, Fowles L, et al. (1997) De novo mutations of the Patched gene in nevoid basal cell carcinoma syndrome help to define the clinical phenotype. Am J Med Genet 73: 304-307.

31. Song YL, Zhang WF, Peng B, Wang CN, Wang Q, et al. (2006) Germline mutations of the PTCH gene in families with odontogenic keratocysts and nevoid basal cell carcinoma syndrome. Tumour Biol 27: 175-180.

32. Romano M, Iacovello D, Cascone NC, Contestabile MT (2011) Identification of a novel mutation in the PTCH gene in a patient with Gorlin-Goltz syndrome with unusual ocular disorders. Eur J Ophthalmol 21: 516-519.

33. Hahn H, Wicking C, Zaphiropoulous PG, Gailani MR, Shanley S, et al. (1996) Mutations of the human homolog of Drosophila patched in the nevoid basal cell carcinoma syndrome. Cell 85: 841-851.

34. Scott A, Strouthidis NG, Robson AG, Forsyth J, Maher ER, et al. (2007) Bilateral epiretinal membranes in Gorlin syndrome associated with a novel PTCH mutation. American Journal of Ophthalmology 143: 346-348.

35. Takahashi C, Kanazawa N, Yoshikawa Y, Yoshikawa R, Saitoh Y, et al. (2009) Germline PTCH1 mutations in Japanese basal cell nevus syndrome patients. J Hum Genet 54: 403-408.

36. Hasenpusch-Theil K, Bataille V, Laehdetie J, Obermayr F, Sampson JR, et al. (1998) Gorlin syndrome: identification of 4 novel germ-line mutations of the human patched (PTCH) gene. Hum Mutat 11: 480.

37. Kansal A, Brueton L, Lahiri A, Lester R (2007) Hypoplastic thumb in Gorlin's syndrome. J Plast Reconstr Aesthet Surg 60: 440-442.

38. Gu XM, Zhao HS, Sun LS, Li TJ (2006) PTCH mutations in sporadic and Gorlin-syndrome-related odontogenic keratocysts. J Dent Res 85: 859-863.

39. Fujii M, Noguchi K, Urade M, Muraki Y, Moridera K, et al. (2011) Novel PTCH1 mutations in Japanese Nevoid basal cell carcinoma syndrome patients: two familial and three sporadic cases including the first Japanese patient with medulloblastoma. J Hum Genet 56: 277-283.

40. Smyth I, Wicking C, Wainwright B, Chenevix-Trench G (1998) The effects of splice site mutations in patients with naevoid basal cell carcinoma syndrome. Hum Genet 102: 598-601.

41. Sasaki R, Saito K, Watanabe Y, Takayama Y, Fujii K, et al. (2009) Nevoid basal cell carcinoma syndrome with cleft lip and palate associated with the novel PTCH gene mutations. J Hum Genet 54: 398-402.

42. Ponti G, Tomasi A, Pastorino L, Ruini C, Guarneri C, et al. (2012) Diagnostic and pathogenetic role of cafe-au-lait macules in nevoid basal cell carcinoma syndrome. Hered Cancer Clin Pract 10: 15.

43. Abe S, Kabashima K, Sakabe J, Shimauchi T, Yan Z, et al. (2008) Coincident two mutations and one single nucleotide polymorphism of the PTCH1 gene in a family with naevoid basal cell carcinoma syndrome. Acta Derm Venereol 88: 635-636.

44. Chung CH, Wong TY, Shieh TY, Shieh DB, Chao SC (2003) Nevoid basal cell carcinoma syndrome - clinical manifestations and mutation analysis of a Taiwanese family. J Formos Med Assoc 102: 793-797.

45. Wilson LC, Ajayi-Obe E, Bernhard B, Maas SM (2006) Patched mutations and hairy skin patches: a new sign in Gorlin syndrome. Am J Med Genet A 140: 2625-2630.

46. Hasenpusch-Theil K, Bataille V, Laehdetie J, Obermayr F, Sampson JR, et al. (1998) Gorlin syndrome: identification of 4 novel germ-line mutations of the human patched (PTCH) gene. Mutations in brief no. 137. Online. Hum Mutat 11: 480.

47. Sasaki R, Miyashita T, Matsumoto N, Fujii K, Saito K, et al. (2010) Multiple keratocystic odontogenic tumors associated with nevoid basal cell carcinoma syndrome having distinct PTCH1 mutations: a case report. Oral Surg Oral Med Oral Pathol Oral Radiol Endod 110: e41-46.

48. Johnson RL, Rothman AL, Xie J, Goodrich LV, Bare JW, et al. (1996) Human homolog of patched, a candidate gene for the basal cell nevus syndrome. Science 272: 1668-1671.

49. Seracchioli R, Bagnoli A, Colombo FM, Missiroli S, Venturoli S (2001) Conservative treatment of recurrent ovarian fibromas in a young patient affected by Gorlin syndrome. Hum Reprod 16: 1261-1263.

50. Garcia de Marcos JA, Dean-Ferrer A, Arroyo Rodriguez S, Calderon-Polanco J, Alamillos Granados FJ, et al. (2009) Basal cell nevus syndrome: clinical and genetic diagnosis. Oral Maxillofac Surg 13: 225-230.

51. Kitsiou-Tzeli S, Willems P, Kosmadaki M, Leze E, Vrettou C, et al. (2011) Nevoid basal carcinoma syndrome (Gorlin syndrome) and pronounced androgenic alopecia in a woman with a novel mutation p.Leu1159fsx32 in the PTCH gene. J Dermatol 38: 1205-1208.

52. Lu Y, Zhu HG, Ye WM, Zhang MB, He D, et al. (2008) A new mutation of PTCH gene in a Chinese family with nevoid basal cell carcinoma syndrome. Chin Med J (Engl) 121: 118-121.

53. Le Brun Keris Y, Jouk PS, Saada-Sebag G, Roux JJ, Mattei B, et al. (2008) Prenatal manifestation in a family affected by nevoid basal cell carcinoma syndrome. Eur J Med Genet 51: 472-478.

54. Kijima C, Miyashita T, Suzuki M, Oka H, Fujii K (2012) Two cases of nevoid basal cell carcinoma syndrome associated with meningioma caused by a PTCH1 or SUFU germline mutation. Fam Cancer 11: 565-570.

55. Musani V, Gorry P, Basta-Juzbasic A, Stipic T, Miklic P, et al. (2006) Mutation in exon 7 of PTCH deregulates SHH/PTCH/SMO signaling: possible linkage to WNT. Int J Mol Med 17: 755-759.

56. Fujii K, Miyashita T, Omata T, Kobayashi K, Takanashi J, et al. (2003) Gorlin syndrome with ulcerative colitis in a Japanese girl. Am J Med Genet A 121A: 65-68.

57. Genevieve D, Walter E, Gorry P, Jacquemont ML, Dupic L, et al. (2005) Gorlin syndrome presenting as prenatal chylothorax in a girl. Prenat Diagn 25: 997-999.

58. Tostar U, Malm CJ, Meis-Kindblom JM, Kindblom LG, Toftgard R, et al. (2006) Deregulation of the hedgehog signalling pathway: a possible role for the PTCH and SUFU genes in human rhabdomyoma and rhabdomyosarcoma development. J Pathol 208: 17-25.

59. Lam CW, Leung CY, Lee KC, Xie J, Lo FM, et al. (2002) Novel mutations in the PATCHED gene in basal cell nevus syndrome. Mol Genet Metab 76: 57-61.

60. Muramatsu S, Suga Y, Mizuno Y, Haseegawa T, Komuro Y, et al. (2005) A Japanese case of naevoid basal cell carcinoma syndrome associated with segmental vitiligo. Br J Dermatol 152: 812-814.

61. Pastorino L, Cusano R, Baldo C, Forzano F, Nasti S, et al. (2005) Nevoid basal cell carcinoma syndrome in infants: Improving diagnosis. Child Care Health and Development 31: 351-354.

62. Yamamoto K, Yoshihashi H, Furuya N, Adachi M, Ito S, et al. (2009) Further delineation of 9q22 deletion syndrome associated with basal cell nevus (Gorlin) syndrome: report of two cases and review of the literature. Congenit Anom (Kyoto) 49: 8-14.

63. Musani V, Cretnik M, Situm M, Basta-Juzbasic A, Levanat S (2009) Gorlin syndrome patient with large deletion in 9q22.32-q22.33 detected by quantitative multiplex fluorescent PCR. Dermatology 219: 111-118.

64. Nowakowska B, Kutkowska-Kazmierczak A, Stankiewicz P, Bocian E, Obersztyn E, et al. (2007) A girl with deletion 9q22.1-q22-32 including the PTCH and ROR2 genes identified by genome-wide array-CGH. American Journal of Medical Genetics Part A 143A: 1885-1889.

65. Fujii K, Ishikawa S, Uchikawa H, Komura D, Shapero MH, et al. (2007) High-density oligonucleotide array with sub-kilobase resolution reveals breakpoint information of submicroscopic deletions in nevoid basal cell carcinoma syndrome. Hum Genet 122: 459-466.

66. Cajaiba MM, Bale AE, Alvarez-Franco M, McNamara J, Reyes-Mugica M (2006) Rhabdomyosarcoma, Wilms tumor, and deletion of the patched gene in Gorlin syndrome. Nat Clin Pract Oncol 3: 575-580.

67. Boonen SE, Stahl D, Kreiborg S, Rosenberg T, Kalscheuer V, et al. (2005) Delineation of an interstitial 9q22 deletion in basal cell nevus syndrome. Am J Med Genet A 132A: 324-328.

68. Midro AT, Panasiuk B, Tumer Z, Stankiewicz P, Silahtaroglu A, et al. (2004) Interstitial deletion 9q22.32-q33.2 associated with additional familial translocation t(9;17)(q34.11;p11.2) in a patient with Gorlin-Goltz syndrome and features of Nail-Patella syndrome. Am J Med Genet A 124A: 179-191.

69. Haniffa MA, Leech SN, Lynch SA, Simpson NB (2004) NBCCS secondary to an interstitial chromosome 9q deletion. Clin Exp Dermatol 29: 542-544.

70. Kosaki R, Nagao K, Kameyama K, Suzuki M, Fujii K, et al. (2012) Heterozygous tandem duplication within the PTCH1 gene results in nevoid basal cell carcinoma syndrome. Am J Med Genet A 158A: 1724-1728.
