# Supplementary material for: PTCH1 Gene Mutations in Keratocystic Odontogenic Tumors: A Study of 43 Chinese Patients and a Systematic Review
Source: PLoS One. 2013 Oct 21;8(10):e77305. doi: 10.1371/journal.pone.0077305 (PMC3804548; doi:10.1371/journal.pone.0077305)
Supplement: Table S2 — Literature review: 17 PTCH1 gene mutations in cases with sporadic KCOTs. (DOCX) [file pone.0077305.s002.docx]

**Table S2.** Literature review: 17 *PTCH1* gene mutations in cases with sporadic KCOTs.

| **Mutation name** | **Patient** | **Nomenclature p.** | **Exon/intron no.** | **Characterization** | **Structure** | **Reference no.** |
| --- | --- | --- | --- | --- | --- | --- |
| **Missense mutations** |  |  |  |  |  |  |
| c.1247C>G | KC17 | p.Thr416Ser | Exon9 | Somatic | ECL1 | [1] |
| c.3265T>C | KC35 | p.Ser1089Pro | Exon19 | Germline | TM10 | [2] |
| c.3277G>C | KC1 | p.Glu1093Arg | Exon19 | Germline | TM10 | [3] |
| c.3913G>T | KC38 | p.Asp1305Tyr | Exon23 | Somatic | C-terminus | [4] |
| **Nonsense mutations** |  |  |  |  |  |  |
| c.403C>T | KC18 | p.Arg135X | Exon3 | Somatic | ECL1 | [1] |
| **Small out-of-frame deletions** |  |  |  |  |  |  |
| c.518_522delAAGCG | KC46 | p.Glu173AlafsX77 | Exon3 | Somatic | ECL1 | [5] |
| c.983delA | KC15 | p.His328LeufsX14 | Exon7 | Somatic | ECL1 | [1] |
| c.1361_1364delGTCT | KC37 | p.Cys454X | Exon10 | Somatic | TM2 | [4] |
| c.1558_1574del17 | KC16 | p.His520ArgfsX44 | Exon11 | Somatic | TM4 | [1] |
| c.2635delG | KC16 | p.Asp879MetfsX24 | Exon16 | Somatic | ECL4 | [1] |
| **Small duplications** |  |  |  |  |  |  |
| c.1325dupT | KC15 | p.Ala443GlyfsX54 | Exon9 | Somatic | TM2 | [1] |
| c.1363_1374dup12 | KC14 | p.Leu455_Leu458dup | Exon10 | Somatic | TM2 | [6,7] |
| c.3068_3074dupGCCTCCG | KC51 | p.Arg1026ProfsX121 | Exon18 | Somatic | ECL4 | [6] |
| c.3124_3129dupCTGTGC | KC36 | p.Val1042_Cys1043dup | Exon18 | Somatic | TM8 | [4] |
| c.3162dupG | KC13 | p.Ile1055AspfsX90 | Exon18 | Somatic | ICL4 | [6,7] |
| c.3162dupG | KC19 | p.Ile1055AspfsX90 | Exon18 | Somatic | ICL4 | [1] |
| c.3295_3300dupCACGTT | KC52 | p.His1099_Val1100dup | Exon19 | Somatic | TM10 | [6] |

**References**

1. Sun LS, Li XF, Li TJ (2008) PTCH1 and SMO gene alterations in keratocystic odontogenic tumors. J Dent Res 87: 575-579.

2. Song YL, Zhang WF, Peng B, Wang CN, Wang Q, et al. (2006) Germline mutations of the PTCH gene in families with odontogenic keratocysts and nevoid basal cell carcinoma syndrome. Tumour Biol 27: 175-180.

3. Wang X, Lu Y, Shen G, Chen W (2011) One germline mutation of PTCH gene in a Chinese family with non-syndromic keratocystic odontogenic tumours. Int J Oral Maxillofac Surg 40: 829-833.

4. Gu XM, Zhao HS, Sun LS, Li TJ (2006) PTCH mutations in sporadic and Gorlin-syndrome-related odontogenic keratocysts. J Dent Res 85: 859-863.

5. Barreto DC, Gomez RS, Bale AE, Boson WL, De Marco L (2000) PTCH gene mutations in odontogenic keratocysts. J Dent Res 79: 1418-1422.

6. Pan S, Li TJ (2009) PTCH1 mutations in odontogenic keratocysts: are they related to epithelial cell proliferation? Oral Oncol 45: 861-865.

7. Pan S, Xu LL, Sun LS, Li TJ (2009) Identification of known and novel PTCH mutations in both syndromic and non-syndromic keratocystic odontogenic tumors. Int J Oral Sci 1: 34-38.
